# The High Plasticity of Nonpathogenic *Mycobacterium brumae* Induces Rapid Changes in Its Lipid Profile during Pellicle Maturation: The Potential of This Bacterium as a Versatile Cell Factory for Lipid Compounds of Therapeutic Interest

**DOI:** 10.3390/ijms232113609

**Published:** 2022-11-06

**Authors:** Víctor Campo-Pérez, Sandra Guallar-Garrido, Marina Luquin, Alejandro Sánchez-Chardi, Esther Julián

**Affiliations:** 1 Departament de Genètica i de Microbiologia, Facultat de Biociències, Universitat Autònoma de Barcelona, 08193 Barcelona, Spain; 2 Bacterial Infections and Antimicrobial Therapy Group, Institute for Bioengineering of Catalonia (IBEC), Baldiri Reixac 15-21, 08028 Barcelona, Spain; 3 Servei de Microscòpia, Universitat Autònoma de Barcelona, 08193 Barcelona, Spain; 4 Departament de Biologia Evolutiva, Ecologia i Ciències Ambientals, Facultat de Biologia, Universitat de Barcelona, 08028 Barcelona, Spain

**Keywords:** cell wall, electron microscopy, lipid inclusions, intrabacterial, mycobacterium, ultrastructural imaging

## Abstract

The immunomodulatory potential of mycobacteria to be used for therapeutic purposes varies by species and culture conditions and is closely related to mycobacterial lipid composition. Although the lipids present in the mycobacterial cell wall are relevant, lipids are mainly stored in intracellular lipid inclusions (ILIs), which have emerged as a crucial structure in understanding mycobacteria-host interaction. Little is known about ILI ultrastructure, production, and composition in nonpathogenic species. In this study, we compared the lipid profiles of the nonpathogenic immunomodulatory agent *Mycobacterium brumae* during pellicle maturation under different culture conditions with qualitative and quantitative approaches by using high-resolution imaging and biochemical and composition analyses to understand ILI dynamics. The results showed wax esters, mainly in early stages of development, and acylglycerols in mature ILI composition, revealing changes in dynamics, amount, and morphometry, depending on pellicle maturation and the culture media used. Low-glycerol cultures induced ILIs with lower molecular weights which were smaller in size in comparison with the ILIs produced in glycerol-enriched media. The data also indicate the simple metabolic plasticity of lipid synthesis in *M. brumae*, as well as its high versatility in generating different lipid profiles. These findings provide an interesting way to enhance the production of key lipid structures via the simple modulation of cell culture conditions.

## 1. Introduction

Lipid profiles in mycobacteria, which show high inter- and intraspecific variability, are modulated by environmental or in vitro culture conditions, being highly dynamic and involving many bacterial compartments. In fact, the main cellular components with high lipid variability are the mycobacterial cell wall and cytoplasm [1]. Mycobacterial lipids are typically associated with the complex mycobacterial envelope, but they are also present in the cytoplasm, where they are stored in intracellular lipid inclusions (ILIs). ILIs are extremely abundant and variable in number and morphometry and are the main players in lipid production in mycobacteria [2,3,4]. ILIs have been described both in pathogenic mycobacteria, such as *Mycobacterium tuberculosis* [5], *Mycobacterium leprae* [6], *Mycobacterium abscessus* [7], and *Mycobacterium kansasii* [8], and in nonpathogenic mycobacteria, such as *Mycobacterium smegmatis* [9] and *Mycobacterium fortuitum* [10]. Despite being commonly found in many species, several crucial aspects of the involvement of ILIs in mycobacterial metabolism and survival remain unknown. ILIs in nonpathogenic mycobacteria have been poorly described, probably because they are considered clinically irrelevant. Specifically, there have been no descriptions of the ILIs in *Mycobacterium brumae* [11], an environmentally nonpathogenic mycobacterium that has been suggested to be efficacious as an immunomodulatory agent for the treatment of non-muscle invasive bladder cancer in preclinical assays [12,13,14].

ILIs are large spheroid aggregates of lipid compounds in the bacterium cytoplasm that act as a carbon reservoir for the obtention of energy through β-oxidation metabolism, since the amount of energy derived from the complete oxidation of fatty acid chains is twice that of a carbohydrate or protein of the same weight [15]. The major and best known lipid storage molecules in ILIs are triacylglycerides (TAGs), although phosphate-rich or wax esters are also present [16,17]. Specifically, TAGs are important in host-pathogen interactions [18] as well as in therapeutic applications of mycobacteria [19]. In this sense, although TAGs seem to be nonreactogenic, they can modify the triggered immune response via a masking phenomenon [20], becoming key compounds for immunomodulatory therapies.

ILIs are highly dynamic structures, and their components can travel and probably be transferred from the cytoplasm to the membrane, helping with membrane maintenance [21]. However, the biosynthesis of ILIs is not well characterized at the molecular level. It has been observed that membrane protein wax ester synthase/acyl-CoA:diacylglycerol acyltransferase allows the formation of small lipid droplets around the cytoplasmatic membrane part of the enzyme. These droplets are then released into the cytoplasm, where the fusion of several of them generates mature ILIs [22]. ILIs have high metabolic plasticity and easily modulate lipid metabolism in these species. Their abundance and composition can vary and largely depends on several parameters, such as the culture medium characteristics, the growing conditions, and the culture growth phase [9]. It has been shown that ILI formation occurs mainly after the exponential growth phase and is especially notable in the presence of a high carbon/nitrogen ratio in the culture medium, with an excess of carbon and a limiting amount of nitrogen being the ideal conditions for ILI formation and accumulation [23].

ILIs are important from a wide range of viewpoints. First, ILIs are strongly related to virulence in pathogenic mycobacteria, e.g., the presence of TAGs inclusions in *M. tuberculosis* has been demonstrated and its development induction has been directly related to the uptaking of host free fatty acids. *M. tuberculosis* growing on fatty acids leads to increased production of phtiocerol dimycocerosate (PDIM) and sulfolipid-1 (SL-1) in its cell wall [24]. *M. tuberculosis* produces enzymes that allow the formation of glycerol intermediates from acquired fatty acids, before esterification of the glycerol with fatty acyl-residues produces TAGs [2,25]. TAGs ILIs of pathogen mycobacteria suppose a source of energy that enable intracellular survival and persistence during the dormant stage, but mycobacteria can also reduce the flux of carbon through the tricarboxylic acid cycle and reduce their growth to gain tolerance to antibiotics [26]. Moreover, pathogenic mycobacteria have a great ability to capture lipid droplets from the cells of their eukaryotic host and use them for their own survival [27,28]. It has been demonstrated that even the growth of *M. tuberculosis* inside adipocytes occurs only after ILIs formation [29]. Therefore, perturbing ILI metabolism is considered a suitable therapeutic target to eradicate mycobacteria since ILIs are a crucial energy source for bacterial cells [9]. Thus, the study of environmental and nonpathogenic mycobacteria that are able to develop abundant ILIs as a survival strategy with competition from other water and soil bacteria under highly dynamic environmental conditions has emerged as an alternative model to avoid the biosafety limitations of pathogenic species [30].

Finally, ILIs can also be an important source of compounds with immunomodulatory activities, since mycobacteria can metabolize stored fatty acids for the synthesis of molecules as phenolic glycolipids (PGL) or PDIM [31]—characteristic in some *M. tuberculosis* complex species—that are exported to the cell wall and have a relevant role in mycobacteria-host interaction [32]. However, morphological and compositional studies in nontuberculous mycobacteria are scarce [9]. In fact, information on the main aspects of ILIs—such as their numbers, nanoscale morphometry, dynamics during different culture growth phases, composition and enhancement for medical applications—is fragmentary or lacking.

To fill these gaps, for the first time a combination of chromatographic and high-resolution microscopy techniques was used to link the ultrastructure and composition of the lipid profile during pellicle maturation in *M. brumae* in four types of culture media differing in amino acid source (L-glutamate or L-asparagine) and amount of carbon source (glycerol). Moreover, a reduced and simplified protocol for electron microscopy was performed to reduce or avoid undesired modifications (artefacts) of the lipids and cellular composition. These qualitative and quantitative approaches permitted us to pursue the following objectives: observe the temporal development lipid profiles dependent on N and/or C sources; detail and quantify ILI ultrastructure and dynamics in different culture media at different times; visualize the differential compositions of ILIs in samples; highlight simple modulation of ILIs; and discuss potential therapeutical applications of the generated lipids with immunomodulatory properties from a nonpathogenic species.

## 2. Results

### 2.1. Pellicle Maturation Induces Large Modifications to the Lipid Composition of M. brumae

**Figure 1:**
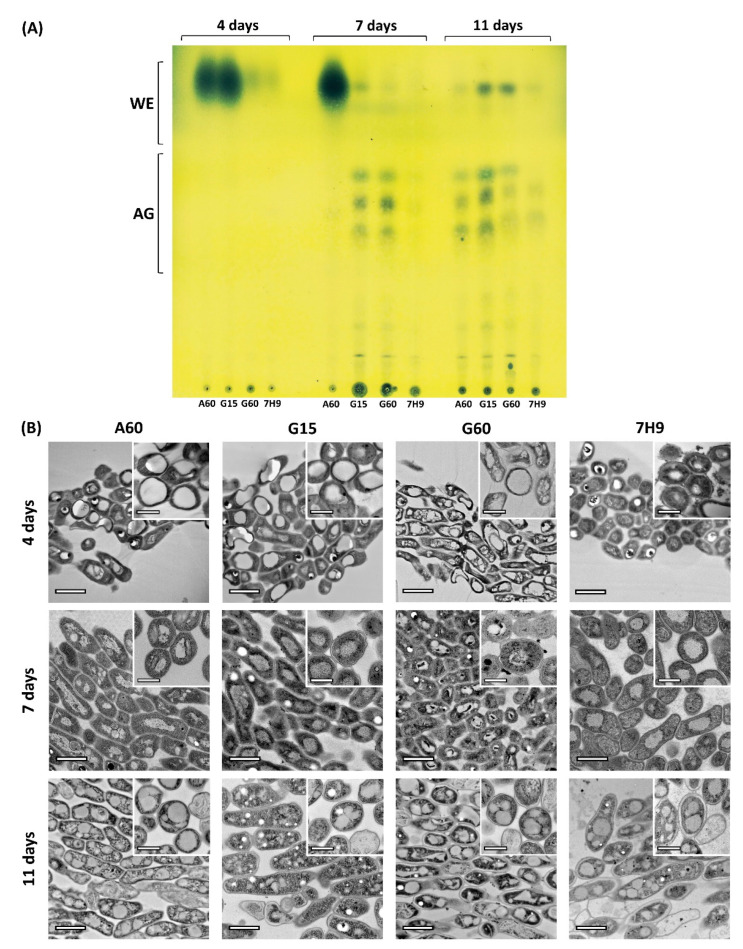
Development of *M. brumae* pellicles and associated lipids during pellicle development in different culture media. (**A**) Thin layer chromatography shows the lipid extract obtained from pellicles grown in each culture medium at 4 (initial), 7 (medium), and 11 (mature) days of development. Lipids were eluted in hexane:diethyl ether:glacial acetic acid (80:20:1, *v*/*v*/*v*) and revealed with 10% molybdophosphoric acid in ethanol. Wax esters (WE) and acylglycerols (AG) were distinguished. (**B**) TEM representative micrographs exhibit the appearance of the pellicle stages at each development state. Scale bars correspond to 1 µm in pellicle general views and 300 nm in bacilli details. Sauton A60, G15, G60 media or Middlebrook 7H9 medium were used to culture mycobacteria. A60 is Sauton containing L-asparagine and 60 mL/L of glycerol (6% *v*/*v*). Sauton G15 and G60 contain L-glutamate and 15 or 60 mL/L of glycerol (1.5% and 6% *v*/*v*), respectively.

Pellicles of *M. brumae* grown in different media were collected to analyse the total mycobacterial lipid components at three maturation stages (4, 7, and 11 days). As Figure 1A shows, the evolution of the mycobacterial lipid content changed during pellicle development and depended on the culture media in which the cells are grown. Cells grown on A60 and G15 media initially (4 days) showed high amounts of wax esters (WEs) that diminished over time. However, acyl glycerides (AGs) increased their concentration on Day 7 (G15) and Day 11 (A60) of development (Figure 1A). In contrast, pellicles grown on G60 and 7H9 media displayed only traces of WEs at 4 days. After this point, G60-grown cells developed a lipid profile containing both WEs and AGs, while small amounts of both lipids were present in 7H9-grown cells at the different time points. When the pellicles had matured, WEs were mainly found in G15- and G60-grown cells, while AGs were predominantly found in cells grown on all Sauton media compared to those grown on 7H9 medium (Figure 1A).

### 2.2. Pellicle Maturation Induces Changes in the Amount of ILIs and Their Ultrastructure in M. brumae Cells

The ultrastructure of the mycobacterial pellicles was analysed at the different stages of growth. At the initial stage (4 days), the pellicles in A60 and G15 media were observed as thin layers formed by a few grouped bacilli presenting notable ILIs, which is in agreement with the large amount of WEs found in these samples. Representative high-resolution images of bacilli sections from the four media at each stage are shown in Figure 1B. At the middle stage (7 days), A60-grown cells continued to contain high amounts of large, round ILIs, while G15- and G60-grown cells showed a lower number of ILIs but abundant unsaturated lipid aggregates externally (shown as electrodense aggregates due to osmium crosslink) around the cell envelope [33,34]). Finally, the cells grown on 7H9 media showed fewer ILIs. These ultrastructural data agree with the observed lipid profile (Figure 1A), displaying the highest number of well-formed large ILIs in high WE-producing medium (A60), a large number of small ILIs in AG-producing media (G15 and G60), and a low number of small ILIs in low WE- and low AG-producing medium (7H9) (Figure 1A,B). These data indicate that diffuse lipid accumulation and a scarce number of small ILIs in the bacterial cytoplasm are related to low lipid production. Mature pellicles (11 days) showed visible ILIs with different sizes, shapes, amounts, and electrodensities in all grown media. Interestingly, all bacilli contained large and numerous ILIs that occupied an important part of the cytoplasm, except those grown in G15 (Figure 1B). This reduction in the number of ILIs from Days 7 to 11 in cells from G15 (the medium that contained the lowest concentration of glycerol) may be related to depletion of the C source, forcing ILIs to be metabolized to obtain energy for cell survival. Overall, combining the information on the lipid profiles and ultrastructures throughout pellicle maturation revealed the rapid adaptation of *M. brumae* cells to resource availability, as they responded by modulating lipid production and storage, which implies important morphological changes.

### 2.3. Ultrastructural Characterization of the ILIs from Mature Pellicles

**Figure 2:**
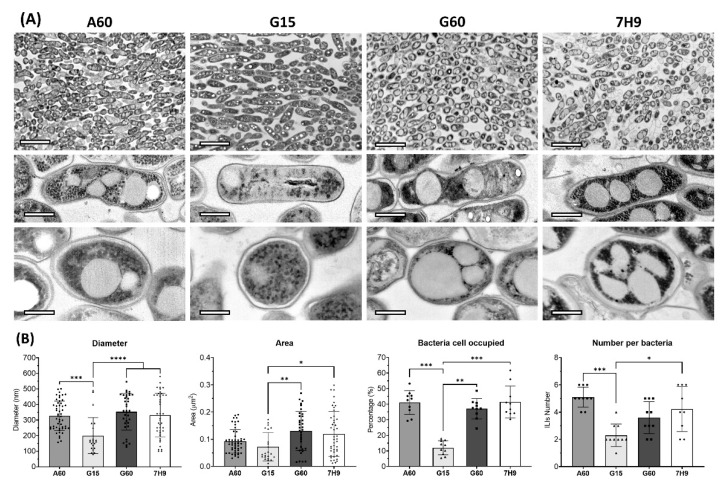
(**A**) TEM representative micrographs of *M. brumae* pellicles developed in different culture media. General perspective and detail of the bacilli in longitudinal and transversal perspective, scale bars correspond to 2 µm, 500 nm, and 250 nm, respectively. (**B**) ILI characterization by quantifying different parameters: diameter, area, percentage of bacteria occupied, and number of ILIs per bacterium using ImageJ version 1.53 (National Institutes of Health, MD, USA) scientific images software. TEM micrographs of ten longitudinally cut bacilli were selected for measurements (*n* = 10), data presented as mean values ± SD statistical analysis using non-parametric multiple comparisons Kruskal-Wallis test, * *p* < 0.05, ** *p* < 0.01, *** *p* < 0.001, **** *p* < 0.0001. Sauton A60, G15 and G60 media, or Middlebrook 7H9 medium were used to culture mycobacteria. A60 is Sauton containing L-asparagine and 60 mL/L of glycerol (6% *v*/*v*). Sauton G15 and G60 contained L-glutamate and 15 or 60 mL/L of glycerol (1.5% and 6% *v*/*v*), respectively.

Representative TEM micrographs at different magnifications showed similar ultrastructures among the bacilli in the four media at the mature stage (Figure 2A). Cells showed an electrodense cytoplasm with evident accumulation of ILIs and a smooth homogeneous electrodensity. However, G15-grown bacilli seem to have a lower number of small ILIs. These differences between the cells grown in different media were highlighted by quantitative analyses of the TEM images. To further characterize the ILIs obtained from the cells in each culture medium, the diameter, area, and percentage of cytoplasm occupied by ILIs, as well as the number of ILIs, were quantified (Figure 2B). The diameters of the ILIs were greater in mycobacterial cells grown in A60, G60, and 7H9 and significantly lower in those grown in G15 medium (see Supplementary Materials Table S1). The percentages of the cytoplasm occupied by ILIs were also different among the pellicles obtained from different media. A significant percentage of the cytoplasm was occupied by ILIs in the cells grown in media with a rich carbon source (A60, G60 and 7H9), reaching values of up to 40%, which is in contrast to the approximate 10% of cytoplasm accumulation in G15-grown cells. Moreover, the lowest number of inclusions per bacterium was detected in G15-grown cells, while A60-grown cells had the highest. In summary, these four parameters showed two clear patterns: first, the ILIs in the cytoplasm of the A60-, G60- and 7H9-grown cells were generally abundant, large, and round; and second, only the G15-grown cells had significantly fewer and smaller ILIs in the cytoplasm.

### 2.4. Compositional Characterization of the ILIs and Cytoplasm from Mature Pellicles

#### 2.4.1. Unsaturated Lipid Distribution

**Figure 3:**
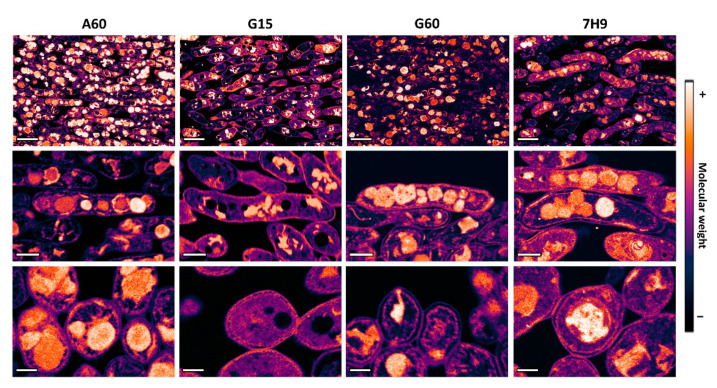
Unsaturated lipids distribution in *M. brumae* cells of mature pellicles grown in the different culture media. Representative FESEM BSE images in pseudocolour showing an overview of cells and details of longitudinal and transversal sections of bacterial cells. Scale bars correspond to 1 µm in general view images, 200 nm in longitudinal and 300 nm in transversal sections of bacilli. Sauton A60, G15, and G60 media, or Middlebrook 7H9 medium were used to culture mycobacteria. A60 is Sauton containing L-asparagine and 60 mL/L of glycerol (6% *v*/*v*). Sauton G15 and G60 contained L-glutamate and 15 or 60 mL/L of glycerol (1.5% and 6% *v*/*v*), respectively.

The FESEM observations of the *M. brumae* pellicle sections fixed with only Os (without staining) permitted analysis of the distribution of this heavy metal linked to unsaturated lipids according to their relative molecular weights in both the ILIs and cytoplasm of the cells. Applying look-up tables (LUTs) to the greyscale BSE micrographs, the resulting pseudocolour images displayed a clear spatial map that revealed the nanoscale distribution of unsaturated lipids in the bacilli (Figure 3). Overall, there were differences in the unsaturated lipid distributions depending on the culture conditions. As reported for ultrastructure, numerous large circular ILIs developed in mature pellicles grown in media with an abundant carbon source (A60, G60 and 7H9), while in the G15 medium (low carbon source) the ILIs were significantly less abundant, small, and presented irregular shapes. Interestingly, the high magnification images showed two important findings, which were especially evidenced in the bacilli from A60 and 7H9 media. In contrast with the homogeneity of the ILIs observed in the ultrastructural images, longitudinal sections of the bacilli revealed high heterogeneity among the ILIs in terms of unsaturated lipid distribution and number visualized as inclusions with different electrodensities (Figure 3). Additionally, nonhomogeneous distributions of unsaturated lipids were found in ILIs, which were especially evident in the images of transversal sections. These inter- and intraspecific variabilities in the molecular weights of the ILIs indicate differences in the numbers of saturated and unsaturated fatty acid chains in the TAGs that form such inclusions. These observed differences are the consequence of the highly dynamic response and high metabolic plasticity of *M. brumae*, which enable triglyceride formation and catalysis from fatty acid chains with different degrees of saturation/unsaturation and lengths attached to glycerol molecules.

#### 2.4.2. Distribution and Quantification of Unsaturated Lipids and Polar Compounds

**Figure 4:**
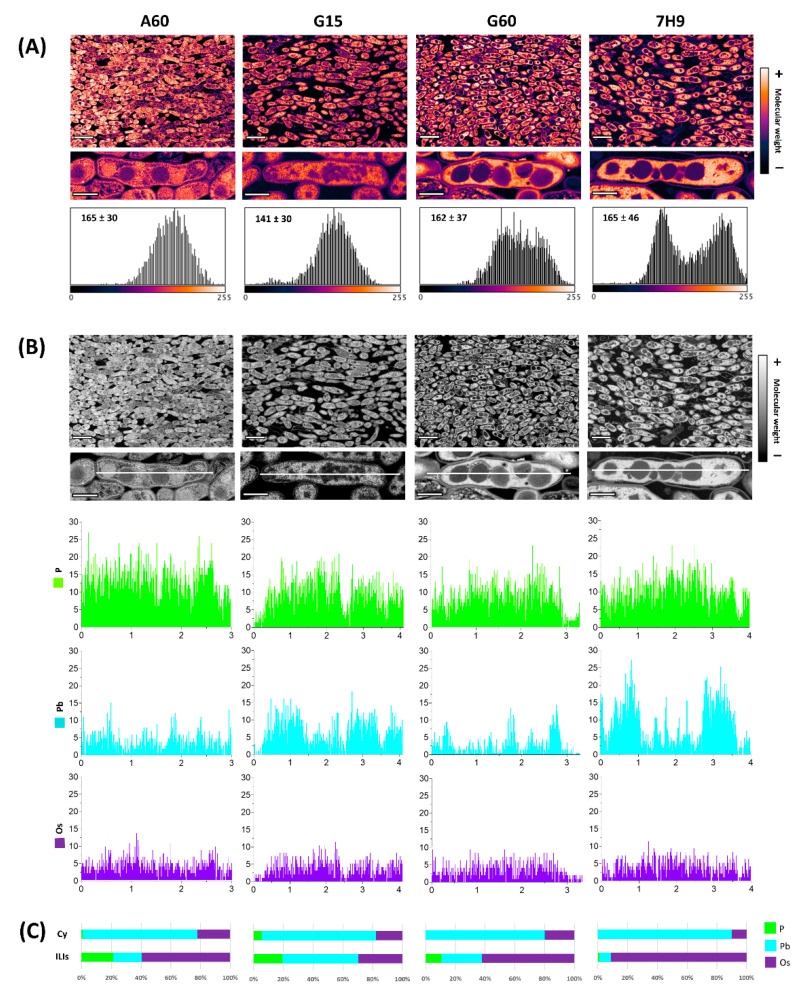
(**A**) FESEM Backscattered pseudocolor micrographs and associated histograms showing molecular weight differences between *M. brumae* pellicles grown in different culture media. Samples fixed with osmium tetroxide and subsequently contrasted with lead citrate. The quantification of molecular weight in histograms showed the colour’s intensities of 10 bacteria of each culture media. (**B**) Compositional characterization of the elements: Phosphorous (P), Lead (Pb) and Osmium (Os) attached to the pellicle by FESEM-EDX line scanning and spot analysis. Scale bars correspond to 2 µm in general and 500 nm in bacilli detail micrographs. (**C**) Percentages of the elements detected in ILIs and cytoplasm correspond with means of values obtained for independent interest points randomly distributed bacilli (*n* = 5) obtained for each culture media. Sauton A60, G15, and G60 media, or Middlebrook 7H9 medium were used to culture mycobacteria. A60 is Sauton containing L-asparagine and 60 mL/L of glycerol (6% *v*/*v*). Sauton G15 and G60 contained L-glutamate and 15 or 60 mL/L of glycerol (1.5% and 6% *v*/*v*), respectively.

In a next step, Os-treated ulltrathin sections were stained with lead citrate to reveal both unsaturated lipids and polar compounds (polysaccharides such as glycogen and proteins of the ribosomes and cytoskeleton, among others) in *M. brumae* cells with mature pellicles. Both BSE and EDX detectors coupled to FESEM were used to analyse the nanoscale distributions of the selected compounds according to their relative molecular weights in the ILIs and cytoplasm. Applying the same LUTs as those used for the BSE images, the obtained pseudocolour micrographs revealed the distribution of unsaturated lipids and polar compounds on the nanoscale (Figure 4). Compared to the images in Figure 3, the higher molecular weights observed as white/orange areas indicated that Pb is mainly linked to the cytoplasm area without ILIs. In contrast, the lower molecular weights observed as darkest/violet areas correspond to ILIs with Os. Interestingly, G15-grown cells presented compounds with a lower molecular weight (mean = 141) than the values of over 160 found for the cells grown on the other types of media (Figure 4A), which could be related to the different metabolic activities of the bacilli depending on the culture conditions.

Finally, indirect detection of biologically relevant compounds and structures with Os (unsaturated lipids) and Pb (polar compounds as proteins and carbohydrates) was used to complement the direct detection of phosphate inclusions and phospholipids with phosphorus (P). The nanoscale distribution of these three elements was analysed using an EDX detector. First, two-dimensional (line scan) analyses of longitudinal sections of bacilli revealed the elemental distribution in the cytoplasm and ILIs in the four types of media (Figure 4B). The detection profiles showed similar distributions and amounts of the three elements in cells grown in all media. However, these elements showed a clear distribution in bacterial cells. Whereas P and Os were bound and found indistinctly throughout all transects and in slightly higher quantities in ILIs, Pb was mainly bound to cytoplasmic areas. Quantification of the 3 elements in the cytoplasm and ILIs revealed interesting differences in ILI composition depending on the culture media. ILIs presented low amounts of phosphates/phospholipids (10–20%) and medium amounts of unsaturated lipids (60%) in A60- and G60-grown cells, whilst similarly low amounts of P and Os were found in G15-grown cells, and no P and a high amount of Os were detected in 7H9-grown cells.

## 3. Discussion

*M. brumae* cell wall thickness and lipid richness conform to a highly hydrophobic surface that allows the formation of a mycobacterial pellicle on the air-water interface of a liquid culture [30]. As they belong to the actinomycetes group, mycobacteria such as *M. brumae* can develop lipid accumulations (ILIs) in their cytoplasm [22]. Clinically relevant ILI formation has been widely described in pathogenic mycobacterial species, especially in *M. tuberculosis*, as their formation is associated with bacterial stress conditions, such as oxidative stress, iron deficiency or exposure to antibiotics, and are used for the survival of the bacterium in the host [35,36]. However, the present study demonstrates for the first time the capacity of *M. brumae*, an environmentally nonpathogenic mycobacterium, to develop large amounts of ILIs when grown under optimal and nonstressful conditions.

In *M. brumae*, ILIs appear in the early stages of pellicle growth and development. Then, they change and evolve in their morphology and composition throughout pellicle maturation, as indicated by lipid extraction, high-resolution imaging, and spatial composition. The rapid accumulation of WEs in cells grown in A60 (at Days 4 and 7) and G15 (at Day 4) media is indicative of the function of these esters being between those of a fatty acid and a fatty alcohol as compounds for the storage of energy and carbon [37]. The characteristics that differentiate these media are that A60 contains asparagine as a nitrogen source as well as 6% glycerol, whilst G15 contains glutamate and only 1.5% glycerol. The production of WEs is also associated with an excess of carbon and nitrogen-limited conditions in early growth rate culture stages [38,39], as is especially clear in the initial stages of G15-grown *M. brumae* pellicles. In A60, asparagine must be hydrolysed by the mycobacteria towards other amino acids such as glutamate and glutamine with relevant roles as initial nitrogen providers in the central mycobacterial nitrogen metabolism [40]. For this reason, mycobacteria grown in asparagine, unlike those grown in glutamate, have a limitation in initial nitrogen providers, and this could hypothetically influence the development of WEs even in the middle phases of pellicle development. In pathogenic species such as *M. tuberculosis*, WE synthesis has also been linked with similar energetic conditions, stressful situations, and dormant state induction [16], which are situations not attributable to *M. brumae*. WEs also act as depots for toxic or useless fatty acids during the growth and storage of evaporation-resistant lipids to maintain a basic water supply [41,42]. WE degradation occurs in growth-accommodating environments and under carbon limitation [43], which could correlate with the medium-mature pellicle development in our study, in which WEs degraded at the same time that the storage of acylglycerols is enhanced. At the mature state of the pellicle (11 days), different acylglycerols compatible with the TAGs in the ILIs predominantly developed during the stationary growth phase, appearing in all *M. brumae* cultures. Comparable temporal variations in the lipid patterns between the exponential and stationary growth phases were reported in *M. tuberculosis* cultures, showing a progressive increase in TAGs throughout growth and a maximum in the stationary phase [44]. Our results are in accordance with these previous data, suggesting that WEs are synthesized and metabolized as an energy source during initial-exponential pellicle development phases, and once the mature pellicle is established and carbon source is limited, mycobacteria cells prioritize the accumulation of TAGs.

Interestingly, species in the phylum Actinobacteria are the only prokaryotic organisms capable of biosynthesizing TAGs [45]. TAG generation in ILIs is directly related to the presence of glycerol through the formation of ester bonds between the hydroxyl groups of glycerol and the carboxyl groups of oleic acid inside cells [45] because both precursors can cross the mycobacterial cell wall by passive diffusion [46]. Moreover, *M. brumae* could use the accumulated TAGs to prevail in harsh, enduring and highly dynamic environments, although TAGs are used as a source of energy in intracellular pathogenic species to survive inside the host [47]. Then, TAGs are hydrolysed to release fatty acids by β-oxidation to obtain the stored energy when required by the cell [48].

The ultrastructural assessment revealed high variability in ILI number, size and amount of cytoplasm occupied in cells grown in all media, and the G15-grown cells showed the lowest values of these analysed ILI parameters. A highly plausible explanation could be that the low amount of glycerol in G15 medium allows *M. brumae* to form ILIs during the earliest stages of pellicle development, but when the carbon source is exhausted at the mature state, the stored lipids are used to survive. These energy requirements produce large morphological changes in ILIs, and they appear as small cumuli with an irregular shape, as observed in the present study (see the microphotographs of the G15-grown bacilli in Figure 2, Figure 3 and Figure 4). Similar temporal ILI size detriment has been previously reported in *M. smegmatis* liquid cultures [9]. These variations are related to the large dynamics of TAG storage, which are enhanced during periods of excess carbon and nitrogen deprivation, modulating both ILI morphology and composition according to medium properties and during pellicle development [49].

Compositional electron microscopy assessment revealed large spatial heterogenicity in TAG inclusion characteristics between cells grown in the same medium and even in the same bacteria, as shown in the BSE images. ILIs are mostly formed by TAGs, but smaller amounts of other components, such as diacylglycerols, free fatty acids, phospholipids, and proteins, have been reported in relation to species and/or media composition [9,42]. These differences inside the ILIs, depending on pellicle maturation, could also be related to variations in glycerol-bound fatty acid chains and their unsaturation grade and length during normal metabolism. Remarkably, the presence of free fatty acids that can bind Os, producing very bright areas in BSE images, has been previously reported in ILIs, especially in cells grown in media containing oleic acid, such as Middlebrook 7H9 [9].

The morphology and composition of the most commonly observed inclusions in *M. brumae* grown in different culture media matched with the ILIs mainly composed of lipidic compounds, including unsaturated lipids that bind Os as detected by EDX. However, apart from ILIs, the detection of polyphosphate (PolyP) granules has also been reported in *M. smegmatis* and pathogenic mycobacteria such as *Mycobacterium avium paratuberculosis* and *M. tuberculosis* [17,50]. Compositional studies with EDX revealed a residual proportion of phosphorus in *M. brumae* inclusions. However, the low amount of this element and the large size of these common inclusions seems indicative of the presence of phospholipids in the inclusions and in the half-unit membrane surrounding them [42]. Additionally, the presence of polyP inclusions cannot be ruled out in *M. brumae* due to the detection of numerous empty cytoplasmic holes with a small diameter, especially in the bacilli grown for 7 and 11 days in G15 medium (Figure 1A). This artefact is compatible with previous observations of polyP inclusions that can be easily lost during conventional processing and sectioning of samples and/or beam damage that occurs during EM visualization, causing them to be observed as empty spheres [51].

Combining the qualitative and quantitative morphological and compositional data from native (lipid profiles) and enhanced nanoscale imaging and composition (modified rapid EM methods), our results revealed the great ability of *M. brumae* to rapidly develop a large amount of ILIs in the cytoplasm when cultured under different culture conditions. Mycobacterial samples visualized by EM are prone to artefacts as a consequence of the thick and complex cell envelope [52], especially in samples with a high density of bacilli, such as a pellicle. Nevertheless, the micrographs obtained by the different techniques used in this study were of high quality, showing the mycobacterial internal structures well. We modified conventional EM methods to allow for the detection of less modified ultrastructures and compositions to obtain more realistic results. The inclusions were perfectly round, and their boundaries and cytoplasm were well defined. In addition, the cell wall appeared conserved, and its thickness allowed some layers to be distinguished and measurements of different physical parameters to be taken as previously described [53,54].

Environmental mycobacteria display rapid adaptation mechanisms to respond to physicochemical changes in highly dynamic true environmental conditions [30]. Such high metabolic plasticity allows lipid inclusion formation from the very early stages of pellicle formation, of which ILIs can occupy up to 40% of the cytoplasm, indicating their importance to the cell. These ILIs are mostly composed of TAGs but have variable characteristics depending on the media in which the mycobacteria grow. Thus, *M. brumae* presents metabolic plasticity that allows the development of numerous large ILIs that may be a competitive advantage.

## 4. Materials and Methods

### 4.1. Bacterial Strains and Culture Conditions

*M. brumae* (American Type Culture Collection, ATCC 51384) was grown on Middlebrook 7H10 agar (Difco Laboratories, Surrey, UK) supplemented with 10% oleic-albumin-dextrose-catalase (OADC) enrichment medium for one week. A suspension similar to the McFarland 1 turbidity patron was performed by collecting a loopful of *M. brumae* isolated colonies in a glass test tube (SciLabware, Stoke-on-Trent, UK) with 0.5 mm diameter glass beads (dDBiolab, Barcelona, Spain) and disaggregating them in phosphate-buffered saline (PBS, pH 7.4) by vortexing for 30–60 s. A volume of 300 µL containing 1.5 × 10^6^ colony forming units (CFUs) of *M. brumae* was used to inoculate glass bottles (dDBiolab) with 50 mL of Middlebrook 7H9 medium (Difco Laboratories) enriched with 10% ADC or one of three different Sauton compositions (A60, G15 and G60) [14]. All Sauton formulas contained 2 g/L citric acid (Fluka Chemika, Steinheim, Germany), 0.5 g/L potassium dihydrogen phosphate (Panreac, Barcelona, Spain), 0.05 g/L ferric ammonium citrate (Sigma-Aldrich, St. Louis, MO, USA), 1 g/L magnesium sulfate heptahydrate (Fluka Chemika), and 1.4 mg/L zinc sulfate (Panreac). In addition, as the nitrogen source, the samples with Sauton G15 and G60 contained 4 g/L L-glutamate (Panreac), and that with Sauton A60 contained 4 g/L L-asparagine (Scharlau, Sentmenat, Spain). The concentrations of glycerol (Panreac), which was used as the carbon source, were 15 mL/L (1.5% *v*/*v*) in G15 medium and 60 mL/L (6% *v*/*v*) in A60 and G60 media.

### 4.2. Lipid Profiles of the Pellicles

*M. brumae* pellicles were collected at 4, 7, and 11 days as the initial, middle, and mature phases of pellicle development, respectively, and exposed to chloroform and methanol (1:2) with constant stirring overnight (ON) as previously reported [55]. Each lipid extract was collected, and the cellular debris was mixed again with chloroform plus methanol (2:1) with constant stirring ON. Both lipid extracts were pooled and analysed by one-dimensional thin-layer chromatography (TLC) on silica gel plates (Merck). The TLC plates were developed with hexane:diethyl ether:glacial acetic acid (80:20:1, *v*/*v*/*v*) and the spots were revealed by spraying 10% molybdophosphoric acid in ethanol followed by heating at 120 °C.

### 4.3. Ultrastructural Morphometry of the Mycobacterial Pellicles

Pellicles at the three growth phases were collected in Nucleopore membranes (Whatman, Maidstone, UK) and placed on aluminium foil in a 6-well culture plate (Nunc; Thermo Fisher Scientific, Waltham, MA, USA) for fixation by osmium vapour with 250 µL of 4% osmium tetroxide (TAAB Lab., Aldermaston, UK). Direct fixation in osmium vapour conserves the lipids, especially those that are unsaturated, in a rapid manner and avoids the use of buffers in conventional aqueous solutions for fixation (aldehydes) and postfixation (osmium), thus eliminating the presence of external elements such as Ca or P. Therefore, the native ultrastructure and composition were more conserved in these *M. brumae* samples than those treated with conventional protocols. Fixed pellicles were placed into Eppendorf tubes, dehydrated in a graded series of ethanol, embedded in Spurr resin (Sigma-Aldrich) and polymerized in silicone moulds at 60 °C for 48 h. Ultrathin sections (70 nm) of selected areas of semithin sections (1 μm) were obtained with an ultramicrotome (UC6, Leica Microsystems GmbH, Wetzlar, Germany) on both carbon-coated Cu and Au grids and contrasted or not depending on the technique for visualization. A set of samples on the Cu grids were routinely contrasted with 2% uranyl acetate for 30 min and 3% lead citrate for 5 min for quantitative and qualitative morphological studies. At least 5 sections from two grids of each sample were visualized by transmission electron microscopy (TEM) (JEM 1400, JEOL Ltd., Tokyo, Japan) equipped with a Erlangshen ES1000W CCD camera (Gatan, Abingdon, UK) operating at 80 kV.

### 4.4. Compositional Analyses of the Mycobacterial Cells

Two sets of Au grids were used for quantitative and qualitative compositional assessments with backscattered electron (BSE) and energy dispersive X-ray (EDX) detectors using field emission scanning electron microscopy (FESEM). One set was observed without contrast to visualize unsaturated lipids through osmium localization because osmium tetroxide covalently links to the double and triple C-C bonds of unsaturated lipids. The other set of grids was observed by adding a contrast agent with lead citrate to visualize both unsaturated lipids with osmium and polar compounds with lead. Samples were visualized with a FESEM instrument (Merlin, Zeiss, Oberkochen, Germany) equipped with both BSE and EDX (Gatan) detectors operating at 2 and 20 kV for BSE and EDX, respectively.

BSE images provide excellent compositional contrast information for qualitative analyses, showing low weight atoms as dark areas and high weight atoms as bright areas (in our case, this technique highlights Os and Pb). To visualize and quantify the spatial distribution of the elements, histograms of pseudocolour micrographs were generated with Fiji-ImageJ applying Image Look-Up Tables (LUTs) [56] using the mean values from 10 randomly distributed bacilli. EDX qualitative and quantitative analyses in both one-dimensional (spot) and two-dimensional (line scan) provided nanoscale localization and quantification of each element of interest. In our case, three elements were selected as tracers of phosphate inclusions (P), unsaturated lipids (Os), and polar compounds (Pb). The percentages of the elements detected in the ILIs and cytoplasm correspond with the mean values obtained for the independent interest points on the randomly distributed bacilli (*n* = 5) obtained for each type of culture media.

### 4.5. Statistical Analyses

Quantitative data are reported as the arithmetic mean ± standard deviation ($\bar{X}$ ± SD). Quantitative data were tested for both normal distribution and homogeneity of variances using Kolmogorov-Smirnov and Levene’s tests, respectively. Comparisons between groups were carried out with the nonparametric multiple comparisons Kruskal-Wallis test, and pairwise comparisons were performed with Student’s *t* tests. All statistical calculations were performed using GraphPad Prism version 8.0.0 (San Diego, CA, USA). Differences were considered significant when *p* ≤ 0.05, and relevant results are labelled in the figures and tables as * *p* ≤ 0.05, ** *p* ≤ 0.01, and *** *p* ≤ 0.001.

## 5. Conclusions

Overall, this study revealed that the relevance of ILIs is not exclusive to survival during infection for pathogenic mycobacteria, and that environmental mycobacteria also develop and use these energy reserves for lipid storage. Rapid and easy modulation of both the amounts and types of lipids in the lipid profile during pellicle maturation in different media can emerge as a potential strategy to produce lipid compounds of interest in *M. brumae* as a cell factory. Finally, the combination of chemical and morphological techniques shown in the present paper is crucial for the correct identification of ILI composition and analysis in mycobacteria.

## Data Availability

Not applicable.

## Supplementary Materials

The following supporting information can be downloaded at: https://www.mdpi.com/article/10.3390/ijms232113609/s1.
