# Supplementary material for: The High Plasticity of Nonpathogenic Mycobacterium brumae Induces Rapid Changes in Its Lipid Profile during Pellicle Maturation: The Potential of This Bacterium as a Versatile Cell Factory for Lipid Compounds of Therapeutic Interest
Source: Int J Mol Sci. 2022 Nov 6;23(21):13609. doi: 10.3390/ijms232113609 (PMC9655737; doi:10.3390/ijms232113609)
Supplement: Supplementary file 1 [file ijms-23-13609-s001.zip › ijms-1951639-supplementary.pdf]

Supplementary Table S1. Measurements of mycobacterial ILIs parameters. Mean and standard deviation (± SD) of diameter, area, number/cell and percentage of *M. brumae* cytoplasm occupied are shown. Values correspond to the analysis of TEM micrographs (n=10) longitudinal sections of bacilli analysed using Image J to accurately take all measurements. Sauton A60, G15, and G60 media, or Middlebrook 7H9 medium were used to culture *M. brumae*. A60 is Sauton containing L-asparagine and 60 mL/L of glycerol (6% v/v). Sauton G15 and G60 contained L-glutamate and 15 or 60 mL/L of glycerol (1.5% and 6% v/v), respectively.

	**Diameter (nm)**	**Area (μm^2)**	**Number / cell (nº)**	**Percentage cell occupied (%)**
**A60**	327.68 ± 94.99	0.096 ± 0.051	5.1 ± 0.738	41.120 ± 7.572
**G15**	200.00 ± 115.56	0.072 ± 0.052	2.3 ± 0.823	12.056 ± 4.547
**G60**	353.05 ± 117.70	0.138 ± 0.081	3.6 ± 1.174	37.071 ± 6.480
**7H9**	345.39 ± 152.97	0.130 ± 0.095	4.6 ± 1.955	41.435 ± 10.184
